# Metformin for the Improvement of Comorbid Depression Symptoms in Diabetic Patients: A Systematic Review

**DOI:** 10.7759/cureus.28609

**Published:** 2022-08-30

**Authors:** Chandani Hamal, Lakshmi Sai Deepak Reddy Velugoti, Godfrey Tabowei, Greeshma N Gaddipati, Maria Mukhtar, Mohammed J Alzubaidee, Raga Sruthi Dwarampudi, Sheena Mathew, Sumahitha Bichenapally, Vahe Khachatryan, Asmaa Muazzam, Lubna Mohammed

**Affiliations:** 1 Internal Medicine/Family Medicine, California Institute of Behavioral Neurosciences & Psychology, Fairfield, USA; 2 Neurology, California Institute of Behavioral Neurosciences & Psychology, Fairfield, USA; 3 Internal Medicine, California Institute of Behavioral Neurosciences & Psychology, Fairfield, USA; 4 Pediatrics, California Institute of Behavioral Neurosciences & Psychology, Fairfield, USA; 5 Pathology Research, California Institute of Behavioral Neurosciences & Psychology, Fairfield, USA

**Keywords:** high blood glucose levels, antidiabetic drugs, metformin, depression, diabetes mellitus

## Abstract

Diabetes mellitus and depression are chronic debilitating disorders and can occur comorbidly. They are thought to be linked not only through environmental and behavioral factors but through molecular mechanisms as well. Antidepressant medication and psychological therapy, standard treatments for depressive symptoms in Type 2 diabetes mellitus, are linked to high rates of treatment failure and non-adherence; therefore, understanding the molecular mechanisms linking diabetes and depression could lead to discovering new targets and developing novel therapeutics. Metformin is considered a first-line anti-diabetic medication for Type 2 diabetes mellitus, and several studies have discussed its antidepressant effect. Metformin is thought to promote neurogenesis, enhance spatial memory function and protect the brain against oxidative imbalance. This systematic review aims to compile information on metformin's effect on depression symptoms and assess current knowledge on the relationship between depression and diabetes. After reviewing several studies, we concluded that metformin might help treat comorbid depression in diabetic patients, but before it can be recommended as a depression medication, more extensive and better-designed trials are needed.

## Introduction and background

Diabetes mellitus is a chronic metabolic disorder characterized by hyperglycemia due to inadequate insulin secretion, insulin resistance, or both [1]. It is divided into two major categories: Type 1 and Type 2 diabetes mellitus. Type 1 diabetes mellitus (T1DM) is an autoimmune disorder in which the pancreatic beta cells responsible for insulin production are destroyed. It usually affects adolescents, but older people can also be affected. Type 2 diabetes mellitus (T2DM) is attributed to insulin resistance in peripheral tissues. It usually affects adults, and its prevalence increases with increasing age. There are four criteria to diagnose diabetes: the fasting plasma glucose (FPG) >125 mg/dl, the two hours plasma glucose level after a 75 g oral glucose tolerance test (OGTT) >200 mg/dl, the random plasma glucose >200 mg/dl with classic symptoms of diabetes such as increased hunger, increased thirst, and frequent urination, or the level of glycated hemoglobin (HbA1C) >6.4% [2]. 

**Figure 1:**
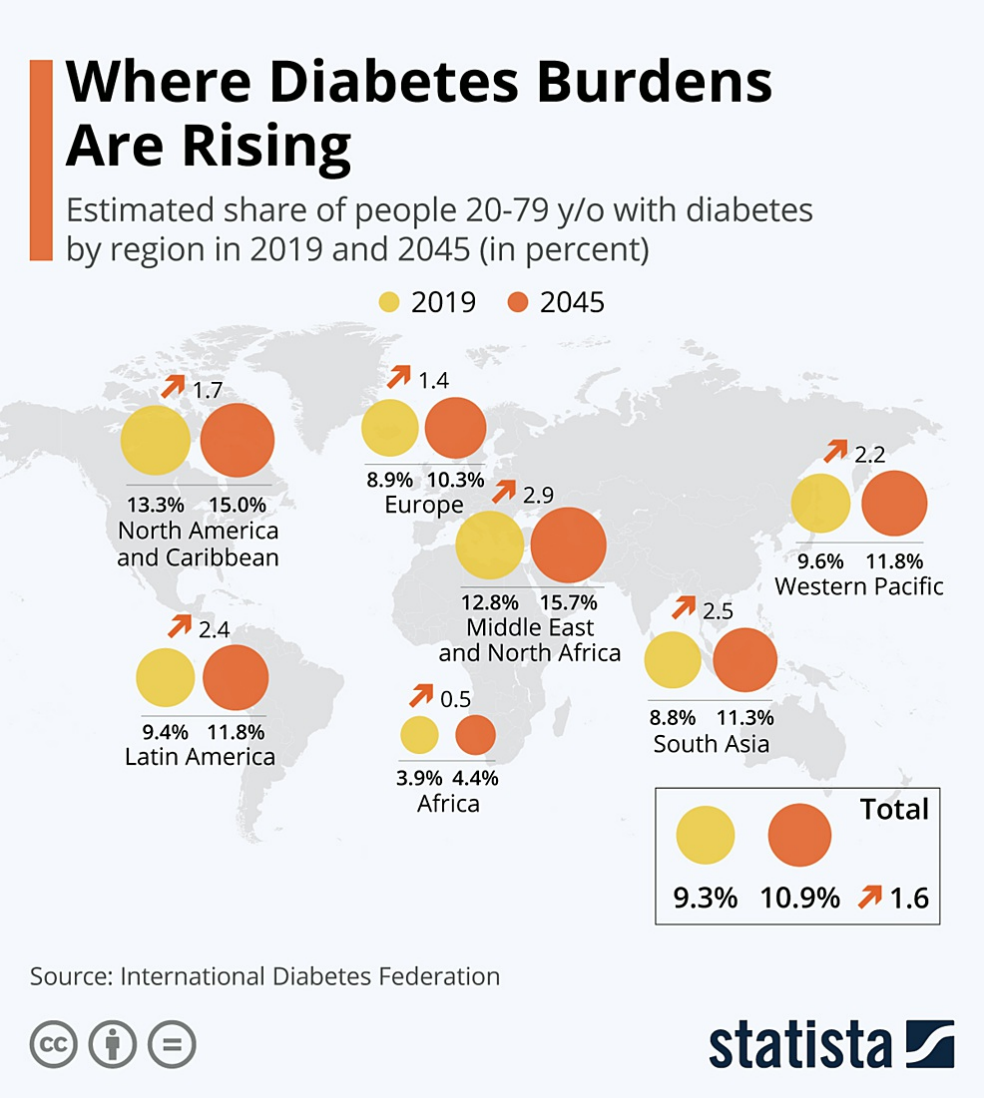
Diabetes burdens in 20-79 year old adults in different continents in 2019 and 2045 (projected). Source: Statista. Permission has been obtained from the original publisher.

Diabetes prevalence has been growing rapidly, with T2DM accounting for more than 95% of people with diabetes [3]. Both T1DM and T2DM are estimated to affect one in 10 adults aged 20 to 79 years or 537 million adults globally, and this number is predicted to rise to 643 million by 2030 and 783 million by 2045 [4]. In 2019, it was the ninth leading cause of death worldwide, with an estimated 1.5 million deaths directly caused by diabetes which increased to 6.7 million deaths by 2021 [3,4]. It has generated at least 966 billion dollars in health expenditure, a 316% increase over the last 15 years [4]. Figure 1 below shows diabetes burdens in 20-79 year-old adults in different continents in 2019 and 2045 (projected), as mentioned by Statista [5].

There are several serious complications associated with diabetes which are divided broadly into microvascular complications (retinopathy, neuropathy, and nephropathy) and macrovascular complications (cardiovascular and cerebrovascular disease) [2]. Furthermore, people with T2DM are at high risk of psychological complications, and previous studies demonstrated that diabetic patients have a higher incidence of depression than the general population [6]. 

Depression is a common mood disorder and is a leading cause of disability worldwide and contributes significantly to the global burden of disease. In 2021, the World Health Organization estimated that 3.8% of the population was affected by depression worldwide [7]. According to the Diagnostic and Statistical Manual of Mental Disorders, Fifth Edition (DSM-5), the diagnosis of a Major Depression Disorder (MDD) requires five or more symptoms to be present within two weeks with one of the symptoms should at least be either a depressed mood or anhedonia (loss of interest or pleasure). The other symptoms of MDD are appetite or weight changes, sleep difficulties, psychomotor agitation or retardation, loss of energy, decreased concentration, feelings of worthlessness or excessive guilt, and suicidality [8]. Subthreshold depression is defined as clinically relevant depressive symptoms that do not meet the MDD criteria [9].

Numerous studies have indicated a greater prevalence of depression in people with diabetes than in non-diabetics. Jones et al. mentioned in their study that up to 30% of people with diabetes mellitus experience clinically significant depression symptoms, and MDD is twice as common in diabetic patients compared to non-diabetics (Odds Ratio (OR): 2.0, 95% Confidence Interval (CI): 1.8-2.2) and patients with MDD (number (n) = 154,366) had a greater risk of T2DM than healthy people (n = 2,098,063; Relative Risk (RR): 1.49; 95 % CI: 1.29-1.72) as seen in a large meta-analysis. Depressive symptoms at baseline were linked to an increase in incident T2DM over a three-year follow-up period in a different longitudinal trial [10]. Depression has also been linked to poor health outcomes in both types of DM and adversely affects diabetes self-care and medication adherence, worsens glycemic control, and lowers the quality of life [11]. 

Understanding this relationship between depression and diabetes has significant implications for discovering new avenues for treatment. Depression may result from comparable environmental factors that regulate glucose metabolism and can also independently influence nutrition and lifestyle decisions, predisposing people to diabetes [9]. Biologically, three pathways have been highlighted for the bidirectional relationship between diabetes and depression. First, hyperglycemia has been found to be associated with depressive symptoms. Secondly, insulin resistance has been suggested as an association between diabetes and depression based on numerous studies. Thirdly, increased inflammation has been discovered both in diabetic and depressed patients, and depressive symptoms have been effectively treated by anti-inflammatory medications. As many anti-diabetic medications affect all three pathways, it has been hypothesized that anti-diabetic medicines might help treat depression in patients with diabetes, possibly reducing polypharmacy [12]. In this systematic review, we will explore whether metformin, which is used as a first-line drug for T2DM, can be used to treat depression symptoms in diabetic patients.

## Review

Methods and materials

This systematic review was performed following the Preferred Reporting Items for Systematic Reviews and Meta-Analyses (PRISMA) 2020 guidelines, in which studies that meet review criteria are examined and included in the study [13]. 

Databases and Search Strategy

The search was conducted using PubMed, PubMed Central (PMC), Google Scholar, Science Direct, and Cochrane Library databases, and articles were retrieved using appropriate keywords, including the medical subject headings (MeSH) strategy. Table 1 demonstrates the databases and search strategy.

**Table 1:** Databases and search strategy Majr: major topic

Databases	Keywords	Filters	Search Result
PubMed	Metformin OR Anti-diabetic medicine OR "Metformin/therapeutic use"[Majr] AND Diabetes Mellitus OR High Blood Glucose OR High Blood Sugar OR ( "Diabetes Mellitus/blood"[Majr] OR "Diabetes Mellitus/complications"[Majr] OR "Diabetes Mellitus/drug therapy"[Majr] ) AND Comorbidity OR "Comorbidity"[Majr] AND Depression OR Sad mood OR Feeling Low OR ( "Depression/blood"[Majr] OR "Depression/drug therapy"[Majr] )	Last Five Years, English, Adults:19+ years, Full Text	1816
Pubmed Central	Metformin, Diabetes, Comorbid depression in adults in the English Language	Last Five Years	134
Google Scholar	Metformin, Diabetes, Comorbid depression in adults in the English Language	2017-2022	27
Science Direct	Metformin and Diabetes and Depression	2017-2022	1129
Cochrane Library	Metformin and Depression	2017-2022	58

All references were grouped using Google Sheets 2022 for duplicate removal. Initially, the records were reviewed based on the titles and abstracts, and irrelevant studies were excluded, followed by retrieval of the full-text articles.

Inclusion and Exclusion criteria

A systematic review was conducted to identify the effectiveness of metformin in treating comorbid depression in diabetic patients more than or equal to 19 years old. To identify relevant literature, electronic databases PubMed, PubMed Central, Google Scholar, Science Direct, and Cochrane Library were searched for English- language publications within the last five years. The choice of studies included were systematic reviews, meta-analysis, cross-sectional, case-control, cohort, literature review, and randomized control trials. Non-English language articles, < 19 years of age, and articles published before 2017 were excluded.

Data Extraction

Two authors conducted the literature review separately, settling disagreements about inclusion through discussions and consensus. After reviewing the titles, abstracts were checked to see if the titles met the requirements for inclusion. Full-text papers were examined from abstracts meeting inclusion criteria, and research still meeting inclusion criteria underwent data extraction.

Risk of Bias in Individual Studies

The remaining full articles were assessed for quality assessment and risk of bias using tools depending on the study type: Systematic reviews and Meta-analyses, Assessment of Multiple Systematic Reviews (AMSTAR); Randomized controlled trials (RCTs), Cochrane Collaboration Risk of Bias Tool (CCRBT); Cross-sectional Studies, Appraisal tool for Cross-Sectional Studies (AXIS); Cohort Studies, Newcastle Ottawa Scale (NOS); and Narrative reviews, Scale for the Assessment of Narrative Review Articles (SANRA) [14-18]. Each assessment tool has its criteria and different scoring system. Each assessment tool required a minimum score of 70% to be acceptable. Table 2 demonstrates the Quality assessment tool and accepted studies for the study.

**Table 2:** Quality assessment of each study AMSTAR: Assessment of Multiple Systematic Reviews, PICO: patient/population, intervention, comparison, and outcomes, RoB: Risk of bias, CCRBT: Cochrane Collaboration Risk of Bias Tool, RCTs: Randomized controlled trials, AXIS: Appraisal tool for Cross-Sectional Studies, NOS: Newcastle Ottawa Scale, SANRA: Scale for the Assessment of Narrative Review Articles

Quality Assessment Tool	Study Type	Items and their characteristics	Total Score	Accepted Score (>70%)	Accepted Studies
AMSTAR [14]	Systematic Review and Meta-analysis	Sixteen components: (1) Did the research questions and inclusion criteria for the review include the components of PICO? (2) Did the report of the review contain an explicit statement that the review methods were established prior to the conduct of the review, and did the report justify any significant deviations from the protocol? (3) Did the review authors explain their selection of the study designs for inclusion in the review? (4) Did the review authors use a comprehensive literature search strategy? (5) Did the review authors perform study selection in duplicate? (6) Did the review authors perform data extraction in duplicate? (7) Did the review authors provide a list of excluded studies and justify the exclusions? (8) Did the review authors describe the included studies in adequate detail? (9) Did the review authors use a satisfactory technique for assessing the risk of bias (RoB) in individual studies that were included in the review? (10) Did the review authors report on the sources of funding for the studies included in the review? (11) If meta-analysis was justified, did the review authors use appropriate methods for the statistical combination of results? (12) If a meta-analysis was performed, did the review authors assess the potential impact of RoB in individual studies on the results of the meta-analysis or other evidence synthesis? (13) Did the review authors account for RoB in individual studies when interpreting/discussing the results of the review? (14) Did the review authors provide a satisfactory explanation for and discussion of any heterogeneity observed in the results of the review? (15) If they performed quantitative synthesis, did the review authors carry out an adequate investigation of publication bias (small study bias) and discuss its likely impact on the results of the review? (16) Did the review authors report any potential sources of conflict of interest, including any funding they received for conducting the review? Rated as YES or NO. Partial Yes was considered as a point.	16	12	Van der Feltz-Cornelis et al. (2021) [9] Moulton et al. (2018) [19]
CCRBT [15]	RCTs	Seven components: 1) random sequence generation (selection bias), 2) allocation concealment (selection bias), 3) selective outcome reporting (reporting bias), 4) other sources of bias, 5) blinding of participants and personnel (performance bias), 6) blinding of outcome assessment (detection bias), and 7) incomplete outcome data (attrition bias). Bias is rated as LOW RISK, HIGH RISK, or UNCLEAR.	7	5	Abdallah et al. (2020) [20] Calkin et al. (2022) [21]
AXIS [16]	Cross-sectional	Five components: 1) Introduction: Were the aims/objectives of the study clear? 2) Methods: Was the study design appropriate for the stated aim(s)? Was the sample size justified? Was the target/reference population clearly defined? (Is it clear who the research was about?) Was the sample frame taken from an appropriate population base so that it closely represented the target/reference population under investigation? Was the selection process likely to select subjects/participants that were representative of the target/reference population under investigation? Were measures undertaken to address and categorize non-responders? Were the risk factor and outcome variables measured appropriate to the aims of the study? Were the risk factor and outcome variables measured correctly using instruments/measurements that had been trialed, piloted, or published previously? Is it clear what was used to determine statistical significance and/or precision estimates? (e.g., p-values, confidence intervals). Were the methods (including statistical methods) sufficiently described to enable them to be repeated? 3) Results: Were the basic data adequately described? Does the response rate raise concerns about non-response bias? If appropriate, was information about non-responders described? Were the results internally consistent? Were the results presented for all the analyses described in the methods? 4) Discussion: Were the authors' discussions and conclusions justified by the results? Were the limitations of the study discussed? 5) Other: Were there any funding sources or conflicts of interest that may affect the authors’ interpretation of the results? Rated as YES or NO. Don’t know/comment was not considered as a point.	20	14	Chin et al. (2020) [22]
NOS [17]	Cohort	Eight components: (1) Representativeness of the exposed cohort (2) Selection of the non-exposed cohort (3) Ascertainment of exposure (4) Demonstration that outcome of interest was not present at the start of the study (5) Comparability of cohorts on the basis of the design or analysis* (6). Assessment of outcome (7) Was follow-up long enough for outcomes to occur (8) Adequacy of follow-up of cohorts Rated as 0, 1, 2. * Maximum of two points are allotted in this category.	8	6	Wium-Andersen et al. (2022) [12] AlHussain et al. (2020) [23] Akimoto et al. (2019) [24] Wang et al. (2017) [25]
SANRA [18]	Literature review	Six components: 1) justification of the article’s importance to the readership 2) statement of concrete aims or formulation of questions 3) description of the literature search 4) referencing 5) scientific reason 6) appropriate presentation of data. Scored as 0, 1, or 2.	12	9	Jones et al. (2021) [10] Grigolon et al. (2019) [26] Woo et al. (2020) [27] Essmat et al. (2020) [28]

Results

**Figure 2:**
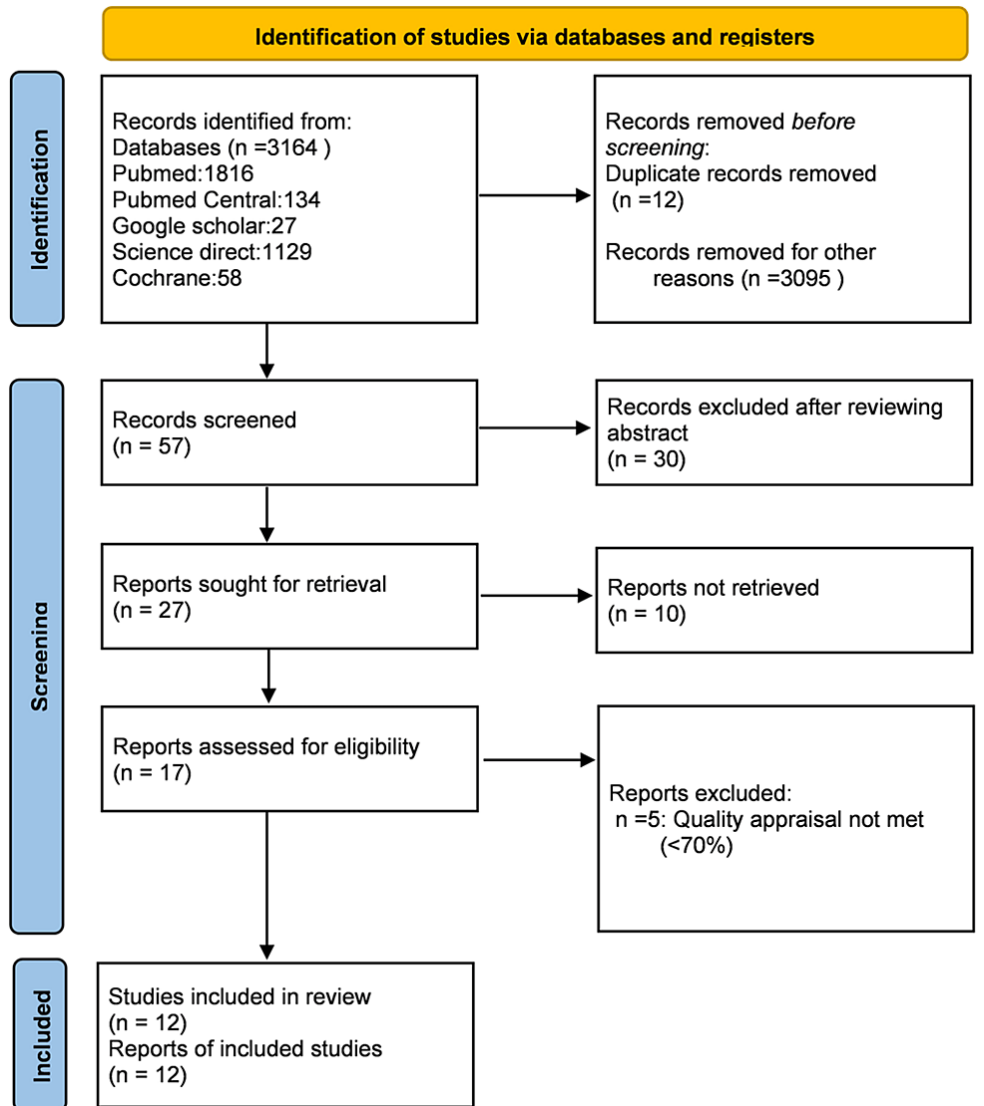
PRISMA flow diagram of literature search PRISMA: Preferred Reporting Items for Systematic Reviews and Meta-Analyses

The search strategy used in this study, as mentioned above in Table 1, included five different databases that yielded 3164 articles, out of which 12 were duplicates and were removed using EndNote, 3095 were removed due to ineligible records, and no automation tools were used. A total of 57 records were screened, out of which 30 were excluded after reviewing the abstract of the records. Ten reports were not retrievable after reading the full articles, and the final screening was down to 17 reports, which were checked for quality and eligibility. After a thorough reading, 12 studies were included in the review. Figure 2 illustrates the PRISMA flow diagram and the search process used in this study [13].

Twelve studies that have met the quality appraisal are included in the systematic review. Table 3 discusses the summary of these studies.

**Table 3:** Summary of studies taken for systematic review SR: Systematic Review, MA: Meta-analysis, CI: Confidence interval, p: p-value, I^2: the level of heterogeneity, r: correlation of coefficient, PHQ-9: Patient Health Questionnaire-9, RCT: Randomized control trial, HbA1C: Hemoglobin A1C, LSMD: least-squares mean difference, BDNF: brain-derived neurotrophic factor, TNF–α: Tissue necrosis factor-alpha, IL-1β: Interleukin-1beta, IL-6: Interleukin-6, IGF-1: Insulin Growth Factor-1, MDA: malondialdehyde, CRP: C-reactive protein, AOR: Adjusted Odds Ratio, DDD: Daily defined doses, BDI: Beck's Depression Inventory, FST: forced swim test

Author/Year of Publication	Study Type	No. of patients (n)	Intervention	Results	Conclusion
Moulton et al. 2018 [19]	SR and MA	n=2638	Metformin	Metformin's impact on depressive symptoms was comparable to that of controls (pooled effect size = +0.32; 95% CI: 0.23 to 0.88; p =.25); however, there was significant heterogeneity (I^2 = 94.2%). Stratified by the kind of controls (placebo or active), metformin was non-significantly better than placebo (pooled effect size 0.49 (95 % C.I. 1.04 to 0.074), p =.089, I^2 = 92.3 %) but worse than active controls (pooled effect size 1.32 (95 % C.I. 0.31-2.34), p 0.001, I^2 = 90.1 %).	In comparison to active controls, mostly pioglitazone, metformin had an overall negative impact on depression symptoms.
Alhussain et al. 2020 [23]	Cohort	n=86	Metformin and Lifestyle Modifications	At the three-month follow-up, patients receiving metformin and lifestyle changes saw a mean decrease of 2.75 points on the PHQ-9 scale, which was used to screen patients for depression, while women receiving only lifestyle changes saw no discernible difference.	Metformin helps in reducing depression symptoms in patients suffering from Polycystic Ovary Syndrome.
Jones et al. 2021 [10]	Literature review	The study mentions an RCT done in 2014 by Guo et al. where n=58	Metformin for 24 weeks	Metformin considerably improved cognitive functions in various domains (i.e., verbal memory, visual memory, attention, and delayed memory). Furthermore, when compared to a placebo, metformin dramatically reduced depression symptoms. Metformin also decreased HbA1C, with a positive correlation between reduction in HbA1C and depressive symptoms in the metformin (r = 0.618, n= 22, p < 0.01).	Metformin helps in reducing depression symptoms comorbid with T2DM through improvements in cognitive performance. Depression symptoms are positively correlated with HbA1C levels.
Abdallah et al. 2020 [20]	RCT	n = 80	fluoxetine (20 mg/day) + metformin (1000 mg/day)	After 12 weeks of therapy, the metformin group exhibited considerably less depression than the placebo group (LSMD: 3.454; 95 % CI: 4.145-2.76). -Compared to the placebo group, the metformin group also exhibited significantly lower levels of TNF–α, IL-1β, IL-6, IGF-1, MDA, and CRP, as well as significantly higher levels of BDNF and serotonin.	Metformin helps in reducing depression symptoms as well as pro-inflammatory cytokines. Metformin also helps in increasing Serotonin and BDNF levels, which plays a vital role in neurogenesis.
Akimoto et al. 2019 [24]	Retrospective cohort study	n=40214	Metformin	The risk of depression was not significantly decreased by the use of metformin (AOR: 0.73; 95% CI: 0.52-1.02; P = .0621).	The use of metformin did not reduce the risk of depression.
Chin et al. 2020 [22]	cross-sectional	n=858	Metformin users Vs Non- metformin users	According to chi-square analysis (P = 0.039), the prevalence of depression in the metformin user group (n = 18 (2.57%)) was lower compared to the nonuser group (n = 9 (6.0%)). These findings remained significant after controlling for age and sex.	The findings show that metformin users had a significantly lower prevalence of depression than nonusers.
Grigolon et al. 2019 [26]	Literature review	The study mentions about: 1) the animal study done by Shivavedi et al. in 2017 2) the study done by Wang et al. in 2012 3) an RCT done in 2014 by Guo et al. where n=58	1) Metformin for 11 days to rats 2) Metformin 3) Metformin for 24 weeks	1) Depressive-like behaviors were significantly decreased in the rats. 2) In cultured neural stem cells, metformin can increase both human and rodent neurogenesis as well as hippocampus neurogenesis and the development of spatial memory. 3) Metformin considerably improved cognitive functions in various domains (i.e., verbal memory, visual memory, attention, and delayed memory). Furthermore, when compared to a placebo, metformin dramatically reduced depression symptoms and also enhanced glucose metabolism in depressed patients with T2DM.	Metformin reduces depression symptoms and helps in neurogenesis and the development of spatial memory.
Wium-Andersen et al. 2022 [12]	Nested case-control study	n=139,996 Metformin use, N (%): 1) Depression cases: 11,178 (16) 2) controls:9259 (13)	Metformin	When compared to diabetic individuals who never used the drug, metformin users had lower odds of developing depression. -Lower doses of metformin (below 1.0 DDD ∼ less than 2g per day) were linked to slightly lower probabilities of depression in comparison to non-users. - Higher doses of metformin (above 1.5 DDD ∼3 g per day) was linked to higher probabilities of depression.	A Lower dose of metformin ( ∼ less than 2 g per day) helps reduce depression compared to a higher dose ( ∼3 g per day).
Woo et al. 2020 [27]	Literature review	The study mentions four studies: 1) RCT done in 2009 by Ackerman et al. (n = 3234 ) 2) an RCT done in 2014 by Guo et al. where n=58 3) study done by Krysiak et al. in 2017 (n=87 ) 4) an RCT done by Jamilian et al. in 2018 (n=60)	1) Metformin For 52 weeks 2) Metformin for 24 weeks 3) Metformin for 26 weeks 4) Metformin for 12 weeks	1) From baseline to endpoint, small but significant BDI reduction occurred with metformin as well as control groups 2) Metformin considerably improved cognitive functions in various domains (i.e., verbal memory, visual memory, attention, and delayed memory). Furthermore, when compared to a placebo, metformin dramatically reduced depression symptoms and also enhances glucose metabolism in depressed patients with T2DM. 3) Compared to the control, metformin significantly decreased the BDI-II score. 4) When compared to metformin, Myo-inositol dramatically decreased the BDI score.	Metformin helps reduce depression symptoms, but when compared to metformin, Myo-inositol is a better option for reducing depression.
Essmat et al. 2020 [28]	Literature review	The study mentions different animal studies	Metformin	Metformin decreased immobility time in FST. Metformin increased serotonin, and BDNF and decreased corticosterone levels.	Metformin help in reducing depression symptoms in animal studied.
Wang et al. 2017 [25]	Cohort	n=41,204	Metformin for nine years	By year nine, metformin was linked to reductions in depression of 0.9% in the healthy class, 5.0% in the high cancer risk class, 2.8% in the high cardiovascular disease risk class, and 15.6% in the high frailty risk class.	Metformin reduced the risk of developing dementia and depression, and several age-related morbidities such as cancer, cardiovascular disease, and frailty.
Calkin et al. 2022 [21]	RCT	n=45	Metformin for 26 weeks	Metformin significantly reduced the Montgomery-Asberg Depression Rating Scale by ≥ 30% in converters (metformin-treated patients who no longer met Insulin Resistant criteria) compared to non-converters.	Patients with Treatment-Resistant Bipolar Disorder who had their Insulin Resistant successfully reversed by metformin saw a statistically significant and clinically significant decrease in their depression rating scale scores.

Discussion

Depression symptoms are two times more common in people with T2DM than in the general population and are linked to an increased risk of diabetic complications and premature death. Antidepressant medication and psychological therapy, standard treatments for depressive symptoms in T2DM, are linked to high rates of treatment failure and non-adherence. Understanding the molecular mechanisms linking DM and depression could lead to discovering new targets and developing novel therapeutics [19]. Several studies have shown the bidirectional relationship between diabetes and depression, and several pathways have been described. In this section, we will discuss some of these pathways and how metformin helps reduce depressive symptoms.

Inflammation

Several studies have shown that circulatory inflammatory markers are a key factor linking T2DM and Depression. The work of Abdallah et al. (2020) showed that inflammation plays a significant role in MDD pathophysiology. They have mentioned in their study that the release of pro-inflammatory cytokines regulates monoamine metabolism, and inflammatory cytokines influence astrocytes leading to a decrease in glutamate reuptake and an increase in glutamate release, as well as a decrease in brain-derived neurotrophic factor (BDNF) production. BDNF affects neuronal integrity and neurogenesis and plays a vital role in depression. Also, patients with MDD have increased pro-inflammatory cytokines such as tumor necrosis factor-alpha (TNF-α) and interleukins (IL) 1β and 6, and the patients experienced an improvement in mood with suppression of cytokine signaling pathways. Through their studies, they found out that Serum levels of TNF-α, IL-1β, IL-6, Insulin Growth Factor-1 (IGF-1), malondialdehyde (MDA), and C-reactive protein (CRP) have been reported to be elevated, and the serum levels of BDNF and serotonin were lower in patients with MDD [20].

The work of Alzoubi et al. (2018) has mentioned several studies supporting inflammation as a shared factor in the bidirectional relationship between T2DM and depression. The studies included are: a Meta-analysis done by Haapakoski et al. (2015), which showed significantly elevated levels of inflammatory biomarkers, such as the CRP and IL-6 in depressed patients; a Meta-analysis by Wang et al. found that elevated levels of inflammatory biomarkers are associated with an increased risk of development of T2DM (RR, 1.26; 95% CI, 1.16-1.37; p-value (p)=0.000), and the work of Au et al. (2014) found out that after approximately six years of follow-up, the Hazard Ratio (HR) for incident T2DM was the highest among participants with elevated depressive symptoms and high CRP levels (age- and sex-adjusted HR, 4.31; 95% CI, 2.70-6.86) [29].

Essmat et al. mentioned that the activation of the hypothalamic-pituitary-adrenal axis (HPAA), production of indoleamine 2, 3-dioxygenase (IDO), and loss of BDNF have all been linked to an increase in neuroinflammatory cytokines and chemokines in the etiology of depression. HPAA activation raises cortisol levels in the blood, which leads to hippocampus shrinkage and depression. IDO, on the other hand, produces depression by activating the tryptophan-kynurenine pathway and lowering serotonin synthesis as a result. As previously stated, BDNF is a neurotrophin vital for neurogenesis, differentiation, and survival.BDNF production in the hippocampus and prefrontal cortex is downregulated either directly by inflammatory cytokines or indirectly by higher glucocorticoid levels due to HPAA activation, resulting in a depressed state in diabetic patients [28]. For physicians and psychiatrists, recognizing biomarkers implicated in MDD pathogenesis is a clinical concern in determining an appropriate treatment plan [20].

Insulin Resistance

Insulin resistance is the next pathway studied linking depression and T2DM. Moulton et al. talked about a cross-sectional study done by Kan et al. (2013), which showed that increased depressive symptoms were consistently associated with higher insulin resistance, even after adjusting confounders [19]. Moulton et al. also talked about another study done by Donath et al. (2014) that showed elevated inflammation is associated with insulin resistance [19]. Grigolon et al. mention studies done by da Silva Dias et al. (2016) and Ma et al. (2015), which showed inflammatory responses are activated in both peripheral and central tissues during the development of plasma insulin resistance, with an increase in IL-1, IL-6, and TNF-α [26].

Some studies talk about brain insulin resistance as a pathway for the development of depression. Jones et al. state that Insulin receptors (IR) are found in brain regions linked to mood disorders, such as the nucleus accumbens, ventral tegmental area, amygdala, and raphe nuclei, where disturbed signaling may play a role in depression [10]. Akimoto et al. talk about mouse models of high-fat diet and T2DM that show signs of insulin resistance in the brain in addition to depressive-like behavior [24]. Brain insulin resistance causes impaired dopamine turnover in mice with a brain-specific deletion of the IR, leading to anxiety and depressive-like behaviors. Hyperinsulinemia caused by peripheral insulin resistance increases lipolysis, creating reactive oxygen species and releasing proinflammatory cytokines resulting in neuroinflammation and insulin resistance in the brain [24]. Woo et al. mention that IR activation, insulin availability, and downstream IR-related processes deficiencies can lead to abnormal IR-mediated functioning and, as a result, various mental diseases, including depression [27].

Hyperglycemia

HbA1C provides a reliable measure of chronic hyperglycemia [30]. Akimoto et al. state that in terms of glycemic control, studies have found that having a high HbA1C increase the risk of depression, and intensive glycemic control has been shown to reduce the risk of diabetic sequelae such as retinopathy, nephropathy, and neuropathy and may also play a key role in preventing the onset of depression. They have found through their study that the HbA1C level was significantly associated with the development of depression (Adjusted Odds Ratio (AOR) for 1.0%: 1.18; 95% Confidence Interval (CI): 1.11-1.26; P < .0001) [24]. Alzoubi et al. describe two studies that show a significant link between depression and hyperglycemia: A meta-analysis of 24 studies done by Lustman et al. (2000) discovered a link between depression and in patients with T1DM and T2DM (p=0.0001), and a study done Richardson et al. (2008) looked at the longitudinal effects of depression on glycemic control in 11,525 veterans with T2DM and found that compared to diabetes patients without depression, diabetes veterans with depression had significantly higher mean HBA1C levels (p=0.008) [29].

Grigolon et al. mentioned that hyperglycemia and hyperinsulinemia are linked to a decrease in serotonergic neurotransmission in the hippocampus and prefrontal cortex regions of the brain, which is crucial for mood and cognition regulation [26]. Persistently high glucose levels increase the formation of advanced glycation products (AGEs), which can stimulate the expression of inflammatory genes, and there is evidence that AGEs accumulate excessively in various moods and other brain illnesses [26]. Essmat et al. state that one of the proposed mechanisms is oxidative stress, which occurs due to hyperglycemia and insulin resistance in people with diabetes. Hyperglycemia reduces the activity of antioxidant enzymes in the brain, accumulating reactive oxygen species (ROS) that lead to apoptotic and necrotic cell death. As a result, cerebral damage and the neurogenesis process are inhibited [28]. The primary regulator of brain inflammation, nuclear factor-kappa B (NF-B), is activated by ROS, which enhances the expression of proinflammatory cytokines such interferon-gamma (IFN), TNF-, cyclooxygenase-2 (COX2), and IL-1 and cause depressive symptoms [26].

Metformin and Its Anti-Diabetic Effect

**Figure 3:**
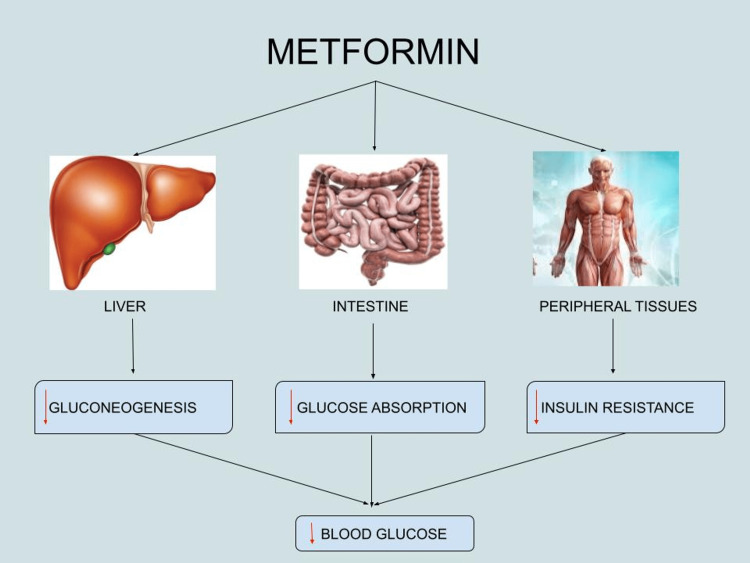
The mechanism of action of metformin as an oral hypoglycemic drug Created by the authors using Google Drawing. Red downward arrows (↓) indicate decrease.

Due to its efficacy in therapy and affordability, metformin is currently the first-line pharmacological agent for the management of T2DM [31]. It is a biguanide that lowers blood glucose levels by decreasing hepatic gluconeogenesis and improving insulin sensitivity via numerous molecular pathways, including mitochondrial control and AMPK activation [27]. It also reduces glucose absorption from the intestine, enhances peripheral glucose uptake, and improves insulin sensitivity [28]. Figure 3, created by the authors, illustrates the mechanism of action of metformin as an oral hypoglycemic drug.

Metformin and Its Anti-Depressant Effect

Metformin's anti-inflammatory, antioxidant, anti-apoptotic, and neuroprotective properties have been shown to have antidepressant benefits in animal models and diabetic individuals with depression. Metformin can inhibit inflammatory cells' adherence to the endothelium. It's also been suggested that metformin can help people with T2DM recover from depression by boosting cognitive performance [20]. 

Experimentally, metformin has been found to work as an antidepressant by modulating 5' adenosine monophosphate- 6 activated protein kinase (AMPK) signaling, a crucial enzyme for maintaining cellular energy homeostasis. Furthermore, a reduction in phosphorylated AMPK (pAMPK) in mice under chronic stress is linked to depression-like behaviors. Metformin inhibits mitochondrial respiratory chain complex I, raising the AMP/ATP ratio and activating AMPK as a result of the shortage of energy and also lowers reactive oxygen species (ROS) levels as well as Nitric Oxide (NO), prostaglandin E2 (PGE2), and pro-inflammatory cytokines (IL-1, IL-6, and TNF-α). Metformin can also activate AMPK by accumulating reactive nitrogen species, stimulating the c-Src/PI3K pathway, and causing molecules to be produced inside the cell, promoting AMPK activation. AMPK activation has been shown to boost BDNF expression via activating the cAMP response element 3 binding protein (CREB) and Akt/glycogen synthase kinase 3 beta (GSK3β) signaling pathways, as well as mTOR signaling, and by regulating DNA hydroxymethylation via the AMPK/Tet2 pathway. In an AMPK-dependent manner, Metformin stimulates Nuclear factor erythroid 2-related factor 2 (Nrf2), a crucial regulator in the brain for limiting inflammatory damage. Its absence may cause more aggressive inflammation by activating the NFκB pathway. Furthermore, metformin's antidepressant-like effect could be linked to the serotonin system and its projection to the hippocampus. Regardless of diabetes status, metformin can suppress the expression of pro-inflammatory cytokines such as IL-1 and IL-6, resulting in enhanced serotonin bioavailability via several routes, including the tryptophan/kynurenine system [27].

**Figure 4:**
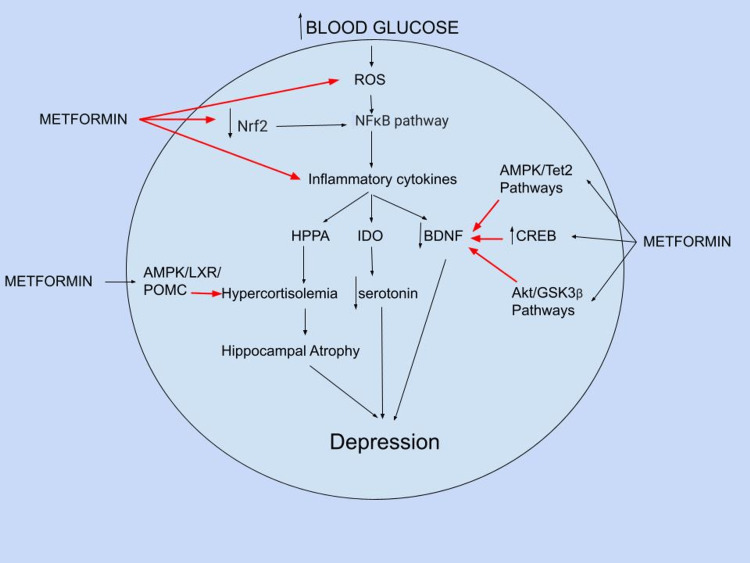
Metformin’s anti-depressant action Created by the author using Google Drawing. Black arrows (→) indicate activation, red arrows (→) indicate inhibition, ROS: reactive oxygen species, Nrf2: Nuclear factor erythroid 2-related factor 2, NFκB: nuclear factor kappa B, HPAA: hypothalamic–pituitary–adrenal axis, IDO: indoleamine 2, 3–5 dioxygenase, BDNF: Brain-derived neurotrophic factor, CREB: cAMP response element 3 binding protein, AMPK: 5' adenosine monophosphate- 6 activated protein kinase; LXRα: liver X receptor alpha; POMC: proopiomelanocortin, GSK3β: glycogen synthase kinase 3 beta.

In conclusion, metformin's potential antidepressant impact suggests that inflammation and oxidative stress play a role in depression via various signaling molecules and pathways, including Nrf2, pro-inflammatory cytokines, and the AMPK/BDNF and NFκB pathways [27]. Moreover, its antidepressant impact may be due to the AMPK/liver X receptor (LXR)/pro-opiomelanocortin (POMC) pathway lowering plasma corticosterone levels and adrenocorticotropic hormone (ACTH) release [28]. Figure 4, created by the authors, demonstrates the overall action of metformin as an anti-depressant.

Limitations

There are some limitations to our study. We chose only published articles in English; some selected studies have a small sample size and cannot represent the whole population. We included studies from 2017 onwards, and there might be some studies before 2017 that might have essential findings. For the future researcher, this study will help answer questions and pave the way to explore further the mechanism for metformin in improving depression in diabetes.

## Conclusions

In conclusion, individuals with diabetes are more likely than those without diabetes to experience depression, and those with depression are more likely to develop diabetes. Environmental and behavioral factors, as well as biological processes, including inflammation, hyperglycemia, and insulin resistance all, demonstrate the bidirectional link between diabetes and depression.

Whether metformin, the first-line treatment for T2DM, has an antidepressant effect is up for debate. On the one hand, certain studies that were part of our systematic review revealed that metformin might lessen the symptoms of depression by lowering hyperglycemia and insulin resistance and by having anti-inflammatory, antioxidant, anti-apoptotic, and neuroprotective characteristics. However, some studies have indicated that it has no beneficial effect on depression. While there is promising evidence for using metformin to treat depression, more extensive and better-designed trials are needed before metformin can be repurposed and recommended as a depression medication.
